# Strong Association of Angiotensin Converting Enzyme-2 Gene Insertion/Deletion Polymorphism with Susceptibility to SARS-CoV-2, Hypertension, Coronary Artery Disease and COVID-19 Disease Mortality

**DOI:** 10.3390/jpm11111098

**Published:** 2021-10-27

**Authors:** Mohammad Muzaffar Mir, Rashid Mir, Mushabab Ayed Abdullah Alghamdi, Badr Abdulmohsin Alsayed, Javed Iqbal Wani, Muffarah Hamid Alharthi, Abdullah M. AL-Shahrani

**Affiliations:** 1 Department of Basic Medical Sciences [Biochemistry], College of Medicine University of Bisha, Bisha 61922, Saudi Arabia; 2 Prince Fahd Bin Sultan Research Chair, Department of MLT, Faculty of Applied Medical Sciences, University of Tabuk, Tabuk 71491, Saudi Arabia; rashid@ut.edu.sa; 3 Department of Internal Medicine, College of Medicine, University of Bisha, Bisha 61922, Saudi Arabia; mualghamdi@ub.edu.sa; 4 Department of Internal Medicine, University of Tabuk, Tabuk 71491, Saudi Arabia; balsayed@ut.edu.sa; 5 Department of Internal Medicine, College of Medicine, King Khalid University, Abha 61421, Saudi Arabia; drjiwani1959@gmail.com; 6 Department of Family medicine, College of Medicine University of Bisha, Bisha 61922, Saudi Arabia; mualharthi@ub.edu.sa (M.H.A.); ab_alshahrani@ub.edu.sa (A.M.A.-S.)

**Keywords:** gene polymorphism, infection, SARS-CoV-2, COVID-19, pathogenesis, ACE1-angiotensin-converting enzyme 1, ACE2-angiotensin-converting enzyme 2, severe acute respiratory syndrome coronavirus 2, susceptibility to SARS-CoV-2

## Abstract

Background: The ongoing outbreak of SARS-CoV-2 represents a significant challenge to international health. Several reports have highlighted the importance of ACE2 on the pathogenesis of COVID-19. The spike protein of SARS-CoV-2 efficiently binds to the angiotensin-converting enzyme 2 (ACE2) receptors and facilitates virus entry into the host cell. In the present study, we hypothesize that a functional insertion/deletion polymorphism-rs4646994 I/D and rs4240157 T > C in the ACE gene could be associated with SARS-CoV-2 infection and mortality. Methodology: This study included 117 consecutive COVID-19 patients and 150 age matched healthy controls (ACE2-rs4646994 I/D) and 100 age matched healthy controls with ACE2 rs4240157 T > C. We used Mutation specific PCR (MSP) for ACE2-rs4646994 I/D genotyping and amplification refractory mutation system (ARMS-PCR) for ACE2 rs4240157 T > C genotyping. Results: Results indicated that there were significant differences in the genotype distributions of ACE2-rs4646994 I/D polymorphisms (*p* < 0.030) and ACE2 rs4240157 T > C between COVID-19 patients and controls (*p*-values < 0.05). Higher frequency of DD genotype (48.71%) and D allele (0.67) was reported in COVID-19 patients than controls. Our results showed that the ACE2-DD genotype was strongly associated with increased COVID-19 severity (OR 2.37 (95%) CI = (1.19–4.70), RR = 1.39 (1.09–1.77), *p* < 0.013) and also a strong association was seen with ACE2-ID genotype with COVID-19 severity (OR 2.20 (95%) CI = (1.08–4.46), *p* < 0.020) in the codominant model. In allelic comparison, the D allele was strongly associated with COVID-19 severity (OR 1.58 (95% CI) (1.11–2.27), RR 1.21 (1.05–1.41) *p* < 0.010). A significant correlation of ACE2-I/D genotypes was reported with Age (*p* < 0.035), T2D (*p* < 0.0013), hypertension (*p* < 0.0031) and coronary artery disease (*p* < 0.0001). Our results indicated ACE2-DD genotype was strongly associated with increased COVID-19 mortality (OR 8.25 (95%) CI = (2.40 to 28.34), *p* < 0.008) and also ACE2-DD + DI genotype was strongly associated with increased COVID-19 mortality with OR 4.74 (95%) CI = (1.5214 to 14.7915), *p* < 0.007. A significant correlation was reported between COVID-19 patients and age matched controls (*p* < 0.0007). Higher frequency of heterozygosity TC (40%) followed by ACE2-CC genotype (24.78%) was reported among COVID-19 patients. Using multivariate analysis, ACE2–CT genotype was strong associated with SARS-CoV-2 severity with an OR 2.18 (95% CI) (1.92–3.99), *p* < 0.010 and also ACE2–CC genotype was linked with COVID-19 severity with an OR 2.66 (95% CI) (1.53–4.62), *p* < 0.005. A significant correlation of ACE2-T > C genotypes was reported with gender (*p* < 0.04), T2D (*p* < 0.035). ACE2-CC genotype was strongly associated with increased COVID-19 mortality OR 3.66 (95%) CI = (1.34 to 9.97), *p* < 0.011 and also ACE2-C allele was associated with COVID-19 mortality OR 2, 01 (1.1761–3.45), *p* < 0.010. Conclusions: It is concluded that ACE-DD genotype and D allele was strongly associated with increased COVID-19 patient severity. In addition, ACE I/D polymorphism were strongly associated with advanced age, diabetes and ischemic heart disease in COVID-19 patients whereas ACE-II genotype was a protective factor against the development of severe COVID-19. ACE2-DD genotype was strongly associated with increased COVID-19 mortality. Additionally, ACE2–CC and CT genotypes were strongly associated with COVID-19 severity. Therefore, our study might be useful for identifying the susceptible population groups for targeted interventions and for making relevant public health policy decisions.

## 1. Introduction

The coronavirus disease, designated as COVID-19 and declared as pandemic by the World Health Organization is caused by severe acute respiratory syndrome coronavirus-2 (SARS-CoV-2) [1,2,3,4]. COVID-19 has infected around 224 million people across the globe and has resulted in more than 4.86 million deaths till date (https://www.worldometers.info/coronavirus, accessed on 12 October 2021). While as the individuals more than 50 years of age were prone to higher severity and mortality of the disease, the common comorbidities included hypertension, obesity, and diabetes [5,6]. COVID-19 has exhibited geographical variations in its spread and mortality and it has caused more morbidity and mortality in Europe and USA [7,8,9].

SARS-CoV-2 is an enveloped RNA virus with three structural protein components which are described as M (membrane), E (envelop) and S (spike) proteins [10,11]. The SARS-COV-2 makes its entry into the host cells through the S proteins. The two subunits of S protein, S1 and S2 are used by the virus to attach to the host cell membrane. The C-terminal domain (CTD) of S1 subunits is vital for the interaction and attachment of S proteins to the protein receptors of the host cell [4,12,13].

Just like the SARS-CoV, the human angiotensin converting enzyme-2 (ACE2) has emerged as the target for the SARS-CoV-2 [14,15]. ACE2 is expressed ubiquitously in different organs of the human body that include heart, lungs, kidneys, intestines and endothelium [16,17,18,19]. ACE2, a type I transmembrane zinc finger glycoprotein, is a mono-carboxypeptidase (with 805 amino acid residues and MW of 100 KD) and converts angiotensin 1 to angiotensin 1-9 [20]. The gene for ACE2, present on chromosome X (Xp22) comprises of 39.98 kb of genomic DNA and has 18 exons [20]. Many ACE2 variants are associated with several common diseases, whose incidence depends on the balance in the renin-angiotensin-aldosterone (RAAS) pathway. Hypertension is associated with rs1514283, rs2074192, rs233575, rs4646155, rs4646176, rs2285666, rs879922, rs2106809, rs4646188, rs4240157, rs4830542, rs2158083, and rs879922 [21,22]. The role of ACE2 is very critical since the inhibition of RAAS pathway leads to upregulation of ACE2 which alleviates ARDS and myocarditis symptoms in COVID-19 patients but at the same time increased ACE2 expression may increase the entry of virus into host cells, complication the overall clinical picture [23].

Geographic distribution of ACE I (insertion in intron 16) allele was summarized by Saab et al. and its frequency increases eastwards and westwards from the Middle East [24]. The genetic polymorphism of ACE2 gene is well known world over with racial and ethnic variations [25,26] having varying influences on the altered functions of RAAS pathway [27]. The polymorphism of ACE2 gene has been associated with hypertension in Chinese [28,29], Canadian [30], Indian [31] and Brazilian populations [32]. The ACE2 polymorphism has been associated with varying degree of disease severity and clinical outcomes of COVID-19, with absence of ACE D/D genotype conferring protection against severe lung injury [33]. The African Americans with high frequency of D allele have depicted higher mortality rates in USA [34,35]. Similarly, higher fatalities have been reported from many parts of Europe (https://ec.europa.eu/eurostat/statistics-explained/index.php/Main_Page, accessed on 12 October 2021). In contrast lower mortality rates have been observed in Asian patients who harbor high frequency of II genotype as compared to DD genotype [36] and (https://www.worldometers.info/coronavirus/coronavirus-death-rate, accessed on 12 October 2021). The D allele results in an increased angiotensin-2 level (as a consequence of increased ACE-1 and decreased ACE-2 levels) that leads to increased microvascular permeability resulting in pulmonary edema and other complications [37,38].

In the current scenario, the knowledge of ACE2 gene polymorphism in different populations assumes a huge significance. However, although there have been many published reports on the ACE2 gene polymorphism from different parts of the globe, we came across only one report from Saudi Arabia suggesting a link between the ACE2-rs4646994 I/D polymorphism and the obesity [39]. To the best of our knowledge, the current study on the genetic polymorphism ACE2-rs4646994 I/D and ACE2 rs4240157 C > T in the ACE2 gene is the first one from Saudi Arabia and was carried out in the COVID-19 patients with different disease severity/outcomes and healthy controls.

## 2. Methodology

### 2.1. Study Population

This population-based case-control study (prospective cohort study) was conducted on COVID-19 patients and 150 age matched healthy controls. COVID-19 laboratory confirmation was defined as a positive result on RT-qPCR (SARS-CoV-2 RT-qPCR detection kit) of nasal and oropharyngeal swab specimens. We selected COVID-19 positive cases confirmed by a real-time reverse transcription polymerase chain reaction (rRT-PCR) test.

This study was a collaborative study. Specimens from positive cases of COVID-19 were collected from different hospitals in Saudi Arabia (Bisha, Abha and Tabuk). The sample collection was conducted between September 2020 and April 2021.

### 2.2. Ethics Approvals

The ethical approvals were obtained from local RELOC committees of College of Medicine, University of Bisha (Ref. No. UBCOM/H-06-BH-087 (05/25)), University of Tabuk (Ref. No: KAEK2020/4/4) and College of Medicine, King Khalid University, Abha (Ref. No. KKU-H-06-B-091) in accordance with human subjects and complied with the principles of the Helsinki Declaration. Informed consent was obtained before collecting samples from all patients and control subjects.

### 2.3. Data Collection

All the subjects were interviewed using a structured questionnaire regarding epidemiological/demographic data, past history of CAD, T2D, and history of addiction particularly smoking, family history of any significant diseases.

#### 2.3.1. Sample Collection from COVID-19 Patients

About 3 mL of peripheral blood sample was collected in an EDTA or Lavender top tube for all COVID-19 patients. The Blood specimens were immediately stored at −20 °C to −30 °C. The COVID-19 patients who’s who oxygen saturation was less than 60 and needed mechanical ventilatory support in ICU were classified as having severe COVID-19 disease.

#### 2.3.2. Sample Collection from Healthy Controls

All healthy age matched controls specimens have been timed around routine blood draws that are part of routine workout, and hence did not require additional phlebotomy and all participants were provided written informed consent form. About 3 mL peripheral blood was collected in EDTA tubes. The Blood specimens were immediately stored at −20 °C to −30 °C.

### 2.4. Genomic DNA Extraction

Genomic DNA was extracted using DNeasy Blood K (Qiagen, Hilden, Germany) as per the manufacturer’s instructions. The extracted DNA was dissolved in nuclease-free water and stored at 4 °C until use. Quality and integrity of DNA were checked by NanoDrop™ (Thermo Scientific, Waltham, MA, USA). All DNA samples from COVID-19 and controls were screened for purity by measuring optical density (OD) at 260 nm (OD_260_) and 280 nm (OD_280_). The λ260/λ280 ratios ranged from 1.83–1.99 indicating good quality DNA.

### 2.5. Angiotensin-Converting Enzyme Genotyping

The Angiotensin-converting enzyme-2 genotyping (ACE2-rs4646994 I/D) was done by mutation specific PCR and amplification refractory mutation system (ARMS-PCR) PCR was used to detect ACE2 rs4240157 T > C genotyping. The ARMS primers were designed by using the Primer3 software as depicted in Table 1.

The PCR was done in a reaction volume of 25 µL containing template DNA (50 ng), Fo—0.25 µL, R0—0.25 µL, RI—0.25 µL, RI—0.25 µL of 25 pmol of each primer and 10 µL from GoTaq^®^ Green Master Mix (cat no M7122) (Promega, Madison, WI, USA). The final volume of 25 µL was adjusted by adding nuclease free double distilled water (ddH_2_O). Finally, 2 µL of DNA was added from each patient. The thermocycling conditions used were: 95 °C for 10 min followed by 40 cycles of 95 °C for 35 s, annealing temperature ACE2-rs4646994 I/D (58 °C) and ACE2 rs4240157 C > T (60 °C) gene polymorphism for 40 s, 72 °C for 43 s followed by the final extension at 72 °C for 10 min.

**Figure 1:**
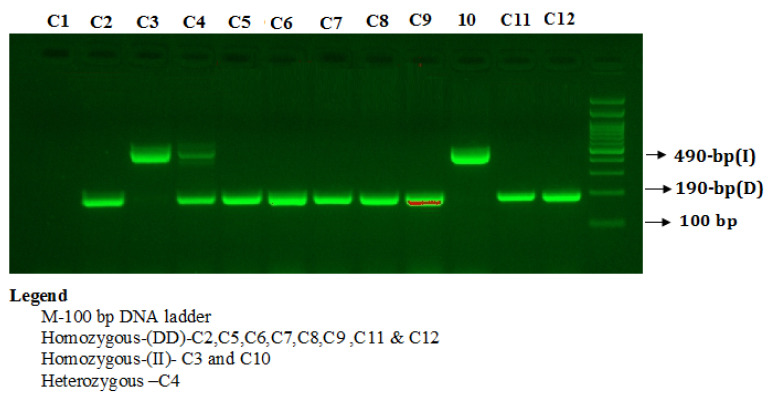
Detection of Angiotensin I-converting enzymes (ACE2) insertion/deletion (I/D) rs4343 gene polymorphism.

PCR products were separated on 2% agarose gel stained with 2 µL of sybre safe stain (Thermo Scientific, Waltham, MA, USA) and visualized on a UV trans illuminator from Bio-Rad (Hercules, CA, USA). The PCR products were electrophoresed on a 2% agarose gel stained with ethidium bromide to visualize three patterns: I/I (490-bp fragment), D/D (190-bp fragment), and I/D (both 490- and 190-bp fragments) as depicted in Figure 1.

**Figure 2:**
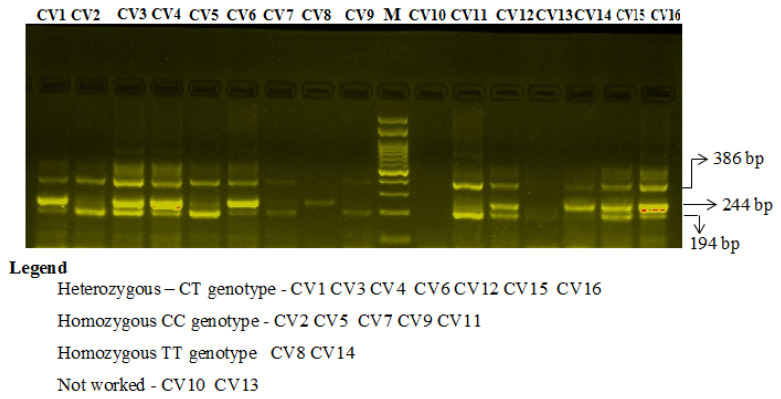
Two percent gel electrophoresis of angiotensin-converting enzyme 2 (ACE2) rs4240157 C/T genotyping by the amplification-refractory mutation system (ARMS) in COVID-19 cases.

Primers FO and RO flank the exon of the ACE2 rs4240157 T > C gene, resulting in a band of 386 bp to act as a control for DNA quality and quantity. Primers FI and RO amplify a wild-type allele (T allele), generating a band of 244 bp, and primers FO and RI generate a band of 194 bp from the mutant allele (C allele) as depicted in Figure 2.

### 2.6. Statistical Analysis

Deviations from Hardy–Weinberg disequilibrium (HWD) were calculated by chi-square (χ^2^) goodness-of-fit test. Group differences were compared using Student’s two-sample t-test or one-way analysis of variance (ANOVA) for continuous variables and chi-squared for categorical variables. Differences in the ACE-rs4646994 I/D, and ACE2 rs4240157 C > T allele and genotype frequencies between groups were evaluated using Chi-square test. The associations between ACE2-rs4646994 I/D, ACE2 rs4240157 C > T genotypes and risk of COVID-19 patients were estimated by computing the odds ratios (ORs), risk ratios (RRs) and risk differences (RDs) with 95% confidence intervals (CIs). Allele frequencies among cases as well as controls were evaluated by using the chi-square Hardy–Weinberg equilibrium (HWE) test. A *p*-value < 0.05 was considered significant. All statistical analyses were performed SPSS 16.0.

## 3. Results

### 3.1. Demographic Features

All demographic features of the 117 consecutive COVID-19 patients are summarized in Table 2. Of 117 consecutive patients, 20 (17.10%) patients were below or equal to 40 years and 97 (82.90%) were above 40 years of age, 85 (72.64%) were males and 32 (27.36%) were females. Among 117 COVID-19 patients, 47 (40.17%) had T2DM, 37 (9.40%) were having hypertension, 11 (9.40%) having CKD and 47 (40.17%) were having low oxygen saturation (<60 mm Hg) at the time of admission (Table 2). 57 (48.71%) stayed more than 30 days in hospital. Regarding treatment of COVID-19 patients 79/117 (67.52%) were treated with antiviral therapy whereas 77/117 (65.81%) were treated with steroid therapy. Clinical outcome of the COVID-19 patients showed that 43/117 (36.75%) patients died and 74/117 (63.24%) were discharged and survived. At the time of diagnosis of COVID-19 patients, 45/117 (38.57%) had high levels of ALT, 104/117 (97.44%) had high levels of CRP and 48/117 (41.3%) had high levels of AST (Table 2).

### 3.2. Healthy Controls

Out of 150 age matched heathy controls 100 (66.67%) were males and 50 (33.33%) were females and 110 (73.33%) were above 40 years of age and 40 (26.67%) were below or equal to 40 years.

### 3.3. Statistical Comparisons between Patients and Controls (p Values) for ACE I/D Genotypes

We found that the ACE I/D genotype (ACE2-rs4646994 I/D) frequency among all the study participants is in compliance to the HWE. The genotype distributions and allele frequencies of the SNPs located in the ACE2-rs4646994 I/D showed no deviation in HWE (all *p* > 0.05) (χ^2^ = 2.24 *p* ≤ 0.13) in the COVID-19 patient group and similarly the genotype distributions and allele frequencies showed that no deviation in HWE (all *p* > 0.05) (χ^2^ = 0.52 *p* ≤ 0.47) in the controls. Thus, we chose 10% samples from normal control group randomly to review genotyping results, showing that the accuracy rate was more than 99%. In COVID-19 patients, the II, DI and DD genotype frequencies were 13.67%, 37.60% and 48.71% respectively whereas in healthy controls II, DI and DD genotype frequencies were 26.66%, 33.34%, and 40% respectively (Table 3). The distribution of ACE2-rs4646994 I/D genotypes observed between COVID-19 patients and healthy controls was significant (*p* < 0.030). Moreover, the frequency of D allele (fD) was found to be significantly higher among COVID-19 patients than in HC (0.67 vs. 0.57) (Table 3).

### 3.4. Multivariate Analysis to Estimate the Association between ACE2-rs4646994 I/D Genotypes and Risk to COVID-19 Severity

A multivariate analysis based on logistic regression like odds ratio (OD) and risk ratio (RR) with 95% confidence intervals (CI) were calculated for each group to estimate the association between ACE2-rs4646994 I/D genotypes and risk to COVID-19 and the data are summarized in Table 4. Our results indicated that in the codominant model, the ACE2-ID genotype was strongly associated with increased COVID-19 severity with OR 2.20 (95%) CI = (1.08–4.46), RR = 1.34 (1.04–1.72), *p* < 0.020. Similarly, ACE2-DD genotype was strongly associated with increased COVID-19 severity with OR 2.37 (95%) CI = (1.19–4.70), RR = 1.39 (1.09–1.77), *p* < 0.013. There is a strong association observed between ACE2–II genotype vs. ACE2-(DI + DD) genotype in dominant inheritance model and leads to increased COVID-19 severity with OR = 2.39 (95%) CI (1.21–4.35), RR = 1.37 (1.11–1.69), *p* < 0.010 (Table 4). In allelic comparison, the D allele was strongly associated with COVID-19 severity with an OR 1.58 (95% CI) (1.11–2.27), RR 1.21 (1.05–1.41) *p*-value = 0.010. No significance was observed in over dominant inheritance model. This result indicates a potential dominant effect of ACE2-DD genotype and D allele on COVID-19 severity in our study group.

### 3.5. Association of ACE2-rs4646994 I/D Genotypes with Comorbid Conditions by COVID-19 Severity

The statistical comparisons (*p*-values) of ACE2-rs4646994 I/D genotypes with comorbid conditions by COVID-19 severity was done by using a multivariate analysis based on logistic regression like odds ratio (OD) and risk ratio (RR) with 95% confidence intervals (CI) (Table 5). Results showed that the there was a significant correlation between the ACE2-rs4646994 I/D genotypes with respect to the age of the COVID-19 patients (*p* < 0.035), COVID-19 patients having T2D (*p* < 0.0013), COVID-19 patients having hypertension (*p* < 0.0031), COVID-19 patients having coronary artery disease (*p* < 0.0001) (Table 5). Similarly, a significant correlation was reported between ACE2 rs4240157 genotypes and COVID-19 cases whose oxygen saturation was less than <60 mm Hg. (*p* < 0.0001). A significant correlation was reported for ACE2 rs4240157 genotype distribution with respect to duration of COVID-19 cases in the hospital (*p* < 0.044). Similarly, in terms of treatment, a significant correlation was reported between antiviral therapy with respect to and ACE2D/I genotype (Table 5).

### 3.6. Correlation of ACE2 I/D Genotypes with Age of COVID-19 Disease Severity

As can be seen in Table 6, our result indicated that in the codominant model, the ACE2-ID heterozygosity genotype (II vs. DI) was strongly associated with increased COVID-19 severity in subjects with age >40 with OR 6.0 (95%) CI = (1.41 to 25.38), RR = 4.12 (1.33 to 12.74), *p* < 0.014. Similarly, ACE2-DD genotype (II vs. DD + DI) was strongly associated with increased COVID-19 severity in subjects with age >40 with OR 3.72 (95%) CI = (1.17 to 11.88), RR = 2.70 (1.21–6.0), *p* < 0.026. In allelic comparison, the D allele was strongly associated with COVID-19 severity with an OR 1.58 (95% CI) (1.11–2.27), RR 1.21 (1.05–1.41) *p*-value = 0.010 (Table 6).

### 3.7. Correlation of ACE2 I/D Genotypes with Mortality of COVID-19 Patients

The results are summarized in Table 7 and indicate that in the codominant model, ACE2-DD genotype (II vs. DD) was strongly associated with increased COVID-19 mortality OR 8.25 (95%) CI = (2.40 to 28.34), RR = 3.26 (1.7894 to 5.9596), *p* < 0.008. Also, in dominant model, ACE2-DD + DI genotype (II vs. DD + DI) was strongly associated with increased COVID-19 mortality with OR 4.74 (95%) CI = (1.5214 to 14.7915), RR = 2.16 (1.4014 to 3.3599), *p* < 0.007. Similarly, in recessive inheritance model, ACE2-DI + II genotype (DD vs. DI + II) was strongly associated with increased COVID-19 mortality with OR 4.0 (95%) CI = (1.7769 to 9.0432), RR = 2.45 (1.4031 to 4.2927), *p* < 0.0008.

### 3.8. Statistical Comparisons between Cases and Controls (p-Values) for ACE2 rs4240157 C > T Genotypes

The genotype distributions and allele frequencies of the ACE2 rs4240157 T > C showed that no deviation was detected in HWE (all *p* > 0.05) (χ^2^ = 3.38 *p* ≤ 0.06) in the controls. Thus, we chose 10% samples from normal control group randomly to review genotyping results, showing that the accuracy rate were more than 99%. The frequency of ACE2 rs4240157 T > C gene variation in COVID-19 cases and controls was TT (35%), TC (40.17%) and CC (24.78%) and controls TT (59%), TC (31%) and CC (10%) respectively (Table 8). The ACE2 rs4240157 T > C gene variation observed between COVID-19 patients and controls was statistically significant (*p* < 0.0007). Moreover, the frequency of C allele was found to be higher among COVID-19 patients than in HC (0.45 vs. 0.26).

### 3.9. Multivariate Analysis to Estimate the Association of ACE2-rs4646994 I/D Genotypes with COVID-19 Severity

A multivariate analysis based on logistic regression like odds ratio (OD) and risk ratio (RR) with 95% confidence intervals (CI) were calculated for each group to estimate the association of ACE2 rs4240157 T > C genotypes with COVID-19 severity (Table 9). In the co-dominant inheritance model, ACE2–CT genotype was associated with COVID-19 severity with an OR 2.18 (95% CI) (1.92–3.99), RR 1.48 (1.07–2.04) *p* < 0.010 as well as ACE2–CC genotype was linked with COVID-19 severity with an OR 2.66 (95% CI) (1.53–4.62), RR 1.68 (1.25–2.26) *p* < 0.005. (Table 9). In case of dominant inheritance model, ACE2-(CT + CC) genotype was associated with COVID-19 severity with an OR 2.66 (95% CI) (1.53–4.62), RR 1.68 (1.25–2.26) *p* < 0.005. In case of recessive inheritance model, ACE2–CC vs. ACE2-(TT + CT) genotype was associated with covid-19 severity with an OR 2.96 (95% CI) (1.36–6.44), RR 1.97 (1.13–3.43) *p* < 0.006. In allelic comparison, the T allele was compared with the C allele. There was a no significant association of C allele with the COVID-19 severity with OR 0.86 (0.59–1.27), RR 0.92 (0.75–1.13), *p*-value = 0.46.

### 3.10. Association of ACE2 rs4240157 T > C Genotypes with Gender and Comorbid Conditions by COVID-19 Severity

The statistical comparisons (*p*-values) of ACE2 rs4240157 T > C genotypes with comorbid conditions by COVID-19 severity was done by using a multivariate analysis based on logistic regression like odds ratio (OD) and risk ratio (RR) with 95% confidence intervals (CI) (Table 10). Results showed that there was a significant correlation between the ACE2 rs4240157 T > C genotypes with respect to the gender of the COVID-19 patients (*p* < 0.046). A significant correlation was reported between the ACE2 rs4240157 T > C genotypes and COVID-19 severity having T2D (*p* < 0.007), hypertension (0.006) and coronary artery disease (*p* < 0.049). Similarly, a significant correlation was reported between ACE2 rs4240157 T > C genotypes and COVID-19 cases whose oxygen saturation was less than <60 mm Hg. (*p* < 0.0001). A significant correlation was reported between steroid therapy and ACE2 rs4240157 T > C genotypes in COVID-19 patients. A significant correlation was also reported between ACE2 rs4240157 T > C genotypes in COVID-19 patients with hypertension (*p* < 0.006) as shown in Table 10.

### 3.11. Correlation of ACE2 rs4240157 T > C Genotypes with Mortality of COVID-19 Patients

Our results indicated that in the codominant model, the ACE2-CC genotype (TT vs. CC) was strongly associated with increased COVID-19 mortality OR 3.66 (95%) CI = (1.34 to 9.97), RR = 1.90 (1.10–3.30), *p* < 0.011 (Table 11). Similarly, in the recessive inheritance model, ACE2-(TT + CT) genotype was strongly associated with increased COVID-19 mortality with OR 5.0 (95%) CI = (2.0640–12.437), RR = 2.10 (1.2567 to 3.53), *p* < 0.004. In allelic comparison, the T allele was compared with the C allele. There was a significant association of C allele with the COVID-19 severity with OR 2, 01 (1.1761–3.45), RR 1.29 (1.05–1.59), *p* < 0.010 (Table 11).

Table 12 summarizes the comparison of ACE2 rs4240157 T > C genotypes with COVID-19 comorbidities. As can be seen, CT genotype is highly significant in T2D OR 3.68 (95% CI 1.495 to 9.064), and RR 1.71 (1.175 to 2.515) in codominant model. In the dominant model CC + CT genotype is also significant in T2D with OR 2.45 (95% CI 1.076 to 5.599) and RR 1.39 (1.048 to 1.844). CT genotype is also very significant with reference to age with *p*-value of 0.014, OR 3.36 (95% CI 1.268 to 8.908) and RR 2.48 (1.155 to 5.353).

## 4. Discussion

Coronaviruses are divided into α, β, γ, and δ genera on the basis of the target host [1,2,3]. Out of these, mammals are infected by α and β-CoV whereas γ and δ-CoV genera tend to infect birds. The current COVID-19 pandemic is found to be caused by SARS-CoV-2 [3,4]. It is a β-CoV that is enveloped, non-segmented and positive-sense RNA virus. Genome sequencing results showed that this newly discovered virus shares 96.2% identity with bat CoV RaTG13 and 79.5% identity with SARS-CoV [10].

### 4.1. Role of RAAS in SARS-CoV-2 Infection

Different ACE genotypes are believed to be associated with development of acute respiratory distress syndrome and several studies have reported that the distribution frequency of ACE insertion/insertion (II) genotype might have a significant influence on COVID-19 mortality [36,40]. It has been reported that ACE2 levels correlate with susceptibility to SARS-CoV-2 infection and men have a higher ACE2 expression in lung than women and Asian people express ACE2 higher than Caucasian and African American populations [41,42].

### 4.2. Association of ACE2-D/I with Gender

Angiotensin converting enzyme is a metallopeptidase that converts angiotensin I (AT-I) to angiotensin II (AT-II) that acts as a vasoconstrictor in addition to its other functions. ACE also degrades bradykinin, a vasodilator substance. ACE2 is also a known SARS virus receptor [14]. Both ACE1 and ACE2 are highly polymorphic. Polymorphisms present in ACE1 are just as important as those in ACE2. As it was reported that the conversion of Ang II to Ang (1–7) by ACE2 is higher in males than females [43] because ACE2 gene is located on the X chromosome and men express more ACE2 than women that depends on the allelic expressions by women, therefore women may be considered of lower sensitivity against the SARS-CoV-2 infection and its lethal effects [43,44].

Several research reports in mice have shown the protective effect of ACE2, whereby severe lung failure is associated with ACE2 downregulation. Immediately after SARS-CoV-2 infection, there is a downregulation of ACE2 [37,38,45]. ACE gene on chromosome 17 consists of 26 exons and 25 introns. A functional insertion-deletion (I/D) polymorphism of 287 bp Alu repeat sequence has been reported in the intron 16 of the ACE gene [44,46]. Jeong et al. have reported that the deletion (D) and insertion allele (I) are associated with an increased and decreased ACE level and enzyme activity, respectively [47]. The ACE2 gene exhibits a high degree of genetic polymorphism [42]. Itoyama et al. [48] reported the correlation of ACE-D allele with the incidence of pneumonia in SARS patients and the death of subjects with acute respiratory distress syndrome. These results tempted us to investigate the role of ACE2 I/D polymorphism in susceptibility to SARS-CoV-2 infection and related mortality. Our results indicated higher overall frequencies of deletion polymorphism in COVID-19 patients (67%) than the insertion polymorphism (33%). The distribution of homozygote insertion (II) genotype is seen to be higher in the healthy population (26.66%) compared with the COVID-19 patients (13.67%). The frequency of heterozygote ID genotype is comparable between COVID-19 patients (37.60%) and control (33.34%) groups. We observed a significant difference in the distribution of homozygote deletion (DD) genotype between COVID-19 cases and controls (*p* < 0.03) (Table 3).

### 4.3. Distribution of ACE2-II, DI and DD Genotypes in the World Populations

The distribution of ACE2-II, DI and DD genotypes in the SARS-CoV-2 patients in the world populations has been summarized in the Table 13 [49]. The frequency of DD genotype in our patient population was high (48.71%) corresponding to the frequency of Brazil (49%), Finland (42.61%), Turkey (34.44%), Iran (37.50%), Spain (37.84%), Russia (39.38%), Italy (37.61%).

Lee et al., reported that populations in France, Italy and Spain have shown high *D* allele frequency between 82% and 87% [50]. Similarly, African Americans in the United States have been reported to have the highest *D* allele frequency (89%) compared with white Americans (69%) [33]. Conversely, populations in East Asian countries such as China, Japan, Taiwan and Korea have a high frequency of the II genotype [51]. Pati et al. [52] reported that the low DD genotype frequency and high II genotype frequency in the ACE gene is strongly correlated with the relatively low mortality rate of COVID-19 among these populations [52]. A higher mortality rate has been shown among the European populations as in the case of black ethnicity in the United States [34]. In the present study, we observed a positive correlation of allele D with infection and mortality rate of COVID-19.

Recent studies have also shown that the ethnic variations of the ACE I/D genotype tend to correlate with the variations in outcomes where populations with a high D genotype frequency tend to experience higher mortality rates [23]. Our results indicated that in the codominant model, the ACE2-DD genotype was strongly associated with increased COVID-19 mortality OR 8.25 (95%) CI = (2.40 to 28.34), *p* < 0.008 (Table 7). Mortality of SARS-CoV-2 subjects is dependent on a wide range of complex phenomena and genetic mutations are one among them [53,54,55,56]. The prevalence of the DD genotype is higher in patients with severe lung infections and is significantly correlated with a high death rate. Mortality related to SARS-CoV-2 infections has been associated to various comorbid conditions such as hypertension, diabetes, hyperlipidemia, coronary artery disease and renal disease [23,57,58,59,60,61,62,63,64]. High producer of ACE, the DD genotype has been associated with susceptibility to hypertension. [34,35,55,62], Type 2 diabetes [59,60], coronary artery disease [57,59] indicating a possible role of ACE I/D polymorphism with SARS-CoV-2 related mortality. Our results reported a significant correlation of ACE2-DD genotypes with advanced age of the COVID-19 patients (*p* < 0.035), with T2D (*p* < 0.0013), hypertension (*p* < 0.0031) and coronary artery disease (*p* < 0.0001) [Table 5]. Our study revealed that age is a significant risk factor for severe disease. Results showed that the there was a significant correlation between the ACE2-rs4646994 I and D genotypes with respect to the Age of the COVID-19 patients (*p* < 0.035). Advanced age is a significant risk factor for mortality from COVID-19, the important reason being the associated increased frequency of comorbid conditions with advancing age [61,63].

### 4.4. Role of ACE2 rs4240157 T > C Gene Polymorphism in COVID-19 Severity

Our results indicated a significant correlation of ACE2 rs4240157 T > C genotypes with the COVID-19 severity among the COVID-19 patients with T2D (*p* < 0.007), with coronary artery disease (*p* < 0.049) and hypertension (*p* < 0.006) (Table 10). Recent studies identified ACE2 polymorphisms that might influence disease severity and indicated that out of 10 studied SNPs, 5 polymorphisms (rs6632680, rs4830965, rs1476524, rs4240157 and rs2048683) indicated an association with higher tissue specific expression of ACE2 resulting in hospitalization whereas rs1548474 polymorphism showed correlation with low tissue expression and lesser severity [23,37,38,39,40]. Variation in circulating ACE2 levels was speculated to be controlled by genetic factors including rs2106809 polymorphism [39,63]. Xiao et al., [64] have earlier reported that a point mutation in the ACE2 gene (Leu584Ala) facilitates entry of SARS-CoV-1 into host cells. Recent studies have proven that several amino acid variants can potentially affect the interaction between the viral S1 protein and ACE2 receptors and thus the level of infection [65]. Different amino acid residues expressed within the ACE2 receptor were observed to be very relevant either by promoting or preventing viral infection. A total of 13 ACE2 polymorphisms enhanced ACE2/S1 recognition, thereby facilitating SARS-CoV-2 infection while as in contrast, 18 SNPs hindered interactions between ACE2 and S1, thereby reduces the infection rate [62]. Patel et al., [59] reported that ACE 2 rs2074192, rs4240157 and rs4646188 variations in T2D exhibited higher risk with hypertension among persons of Australian descent. ACE2 s4240157 (*p* < 0.001) and rs4646156 (*p* < 0.037) are correlated with increased blood pressure [64]. Our results reported higher frequency of heterozygosity among SARS-CoV-2 cases of ACE2-rs4240157 TC genotypes (40.17%) followed by TT genotypes (35%), and CC genotypes (24.78%) respectively [Table 8].

In our study, the ACE2 rs4240157 T > C gene polymorphism observed between COVID-19 patients and controls was statistically significant (*p* < 0.0007). Wooster et al., [55] reported that ACE2 rs4240157 polymorphisms is associated with COVID-19 disease severity as it might be inducing higher tissue specific expression of ACE2 resulting in the hospitalization of COVID-19 patients. Pouladi et al., [62] reported the association ACE2 rs4240157 T > C gene polymorphism with hypertension and other related heart diseases. Our results indicated ACE2-CT genotype association with COVID-19 severity with an OR 2.18 (95% CI) (1.92–3.99), *p* < 0.010 and also ACE2-CC genotype was linked with COVID-19 severity with an OR 2.66 (95% CI) (1.53–4.62), *p* < 0.005 (Table 4). We observed that ACE2-CC genotype was associated with increased COVID-19 mortality with OR 3.66 (95%) CI = (1.34 to 9.97), *p* < 0.011 (Table 10). Similarly, in recessive inheritance model, ACE2-(TT + CT) genotype was associated with increased COVID-19 mortality with OR 5.0 (95%) CI = (2.0640–12.437), *p* < 0.004.

Despite the relatively small sample size, our results indicate that there is a close association between the ACE I/D gene polymorphism with clinical severity of COVID-19 disease. In addition, advanced age, coronary artery disease and diabetes were independent risk factors for the development of severe COVID-19 disease and mortality. The ACE-II genotype was a protective factor against the development of severe COVID-19 disease. Since this study is a first of its kind from Saudi Arabia, we believe that the relation of ACE2 DD genotype with the disease severity and clinical outcome in COVID-19 patients should be further investigated with more extensive studies. However, none of the studied SNPs are common in the general population. This finding might help in the recognition of people less and more prone to COVID-19.

## 5. Conclusions

It is concluded that ACE-DD genotype and D allele was strongly associated with increased COVID-19 disease severity. In addition, ACE I/D polymorphisms were strongly associated with advanced age, diabetes and coronary artery disease in COVID-19 patients whereas ACE-II genotype was a protective factor against the development of severe COVID-19. ACE2-DD genotype was strongly associated with increased SARS-CoV-2 mortality. Additionally, ACE2-CC and CT genotypes were strongly associated with SARS-CoV-2 severity. Therefore, our study might be useful for identifying the susceptible population groups for targeted therapeutic interventions and for making relevant public health policy decisions. The limitations of the study include a relatively smaller sample size of 117 patients and the fact that COVID-19 patients without symptoms were not included in the study.

## Figures and Tables

**Table 1:** Primers for ACE2 gene polymorphisms.

Direction	Primer Sequence	PCR Product	Annealing Temperature
ARMS-PCR primers of ACE2-rs4646994 I/D gene polymorphism			
ACE-F	5′-CTGGAGACCACTCCCATCCTTTCT-3′	490-bp (II)	58 °C
ACE-R	5′-GATGTGGCCATCACATTCGTCAGAT-3′.	190-bp(DD)	
ARMS-PCR primers of ACE2 rs4240157 T > C gene polymorphism			
ACE2 Fo	GCTGAGTTCTCAAAATAATGCCATAGAT	386 bp	60 °C
ACE2 Ro	GCATTTCTTTCCAATCATTAAGAGTTCA		
ACE2 FI-T	GCCTCAGAACATTACAGAATCAACCT	244 bp	
ACE2 RI-C	GAGGGTTGGTAAATAGTGTTCAGTGG	194 bp	

**Table 2:** Baseline characteristics of the COVID-19 patients. T2D—type2 diabetes; CKD—chronic kidney disease; CAD—coronary artery disease; SpO_2_—partial pressure of oxygen; ALT—alanine transaminase; CRP—C reactive protein; AST—aspartate transaminase.

Clinical Feature	Variable	N = 117	%
Age	>40	97	82.90
	<40	20	17.09
Gender	Males	85	72.64
	Females	32	27.36
T2D	Yes	47	40.17
	No	70	59.83
CKD	Yes	11	9.40
	No	106	90.60
Hypertension	Yes	37	31.62
	No	80	68.37
CAD	Yes	17	14.53
	No	100	85.47
Oxygen saturation	<60	47	40.17
	>80	70	59.83
Duration in hospital	>30	57	48.71
	<30	60	51.29
ALT	<36	72	61.53
	>36	45	38.57
CRP	0.8	13	2.56
	0.8 high	104	97.44
AST	<40	69	58.97
	>40	48	41.3
Antiviral therapy	Yes	79	67.52
	No	38	32.48
Steroids therapy	Yes	77	65.81
	No	40	34.19
Survival	Yes	43	36.75
	No	74	63.24

**Table 3:** Association of ACE2-rs4646994 I/D genotypes in COVID-19 severity.

Subjects	N	II%	DI%	DD%	fD	χ^2^	I	D	*p*-Value
Cases	117	16 (13.67)	44 (37.60)	57 (48.71)	2	6.77	0.33	0.67	0.03
Controls	150	40 (26.66)	50 (33.34)	60 (40)			0.43	0.57	

**Table 4:** Multivariate and ordinal regression risk factor analysis for COVID-19 severity with ACE2-rs4646994 I/D genotypes.

Genotypes	Healthy Controls	COVID-19 Cases	Odd Ratio OR (95% CI)	Risk Ratio RR (95% CI)	*p*-Value	Status
	(N = 150)	(N = 117)				
Codominant						
ACE2-II	40	16	1 (ref.)	1 (ref.)		
ACE2-ID	50	44	2.20 (1.08–4.46)	1.34 (1.04–1.72)	0.02	S
ACE2-DD	60	57	2.37 (1.19–4.70)	1.39 (1.09–1.77)	0.013	S
Dominant						
ACE2-II	40	16	1 (ref.)	1 (ref.)		
ACE2-(DI + DD)	110	101	2.39 (1.21–4.35)	1.37 (1.11–1.69)	0.010	S
Recessive						
ACE2-(II + DI)	90	60	1 (ref.)	1 (ref.)		
ACE2-DD	60	57	1.42 (0.87–2.32)	1.17 (0.93–1.45)	0.15	NS
Allele						
ACE2-I	130	76	1 (ref.)	1 (ref.)		
ACE2-D	170	158	1.58 (1.11–2.27)	1.21 (1.05–1.41)	0.010	S
Over dominant						
ACE2-II + DD	90	73	1 (ref.)	1 (ref.)		
ACE2-ID	50	44	1.08 (0.65–1.80)	1.03 (0.82–1.31)	0.75	NS

**Table 5:** Statistical comparisons (*p*-values) of ACE2-rs4646994 I/D genotypes with comorbid conditions and some clinical parameters with COVID-19 disease severity. T2D—type2 diabetes; CKD—chronic kidney disease; CAD—coronary artery disease; SpO_2_—partial pressure of oxygen; ALT—alanine transaminase; CRP—C reactive protein; AST—aspartate transaminase.

Clinical Feature	Variable	N = 117	II	DI	DD	fD	χ^2^	*p*
Age	>40	97	10	40	47	2	6.7	0.035
	<40	20	6	04	10			
Gender	Males	85	12	30	43	2	0.71	0.70
	Females	32	04	14	14			
T2D	Yes	47	5	27	15	2	13.3	0.0013
	No	70	11	17	42			
CKD	Yes	11	3	2	6	2	2.94	0.222
	No	106	13	42	51			
Hypertension	Yes	37	10	17	10	2	13.28	0.0031
	No	80	06	27	47			
CAD	Yes	17	10	02	05	2	34.22	0.0001
	No	100	06	42	52			
Oxygen saturation [SpO_2,_ mm Hg]	<60	47	06	05	36	2	27.1	0.0001
	>80	70	10	39	21			
Duration in hospital	>30	57	10	15	32	2	6.24	0.044
	<30	60	06	29	25			
ALT	<36	72	10	24	38	2	1.55	0.46
	>36	45	06	20	19			
CRP	<0.8	13	0	4	9	2	3.44	0.17
	>0.8 high	104	16	40	48			
AST	<40	69	10	19	40	2	7.57	0.022
	>40	48	06	25	17			
Antiviral therapy	Yes	79	08	21	50	2	20.1	0.0001
	No	38	08	23	07			
Steroids therapy	Yes	77	11	29	37	2	0.08	0.96
	No	40	05	15	20			
Survival	Death occurred	43	11	20	12	2	13.23	0.0007
	Survived	74	05	24	45			

**Table 6:** Statistical comparisons (*p*-values) of ACE2-rs4646994 I/D genotypes with age of COVID-19 severity.

Age	<40	>40	Odd Ratio (95% CI)	Risk Ratio (95% CI)	*p*-Value
	N = 20	N = 97			
Codominant inheritance model					
II	06	10	Ref	Ref	
DI	04	40	6.0 (1.41 to 25.38)	4.12 (1.33 to 12.74)	0.0149
DD	10	47	2.82 (0.83 to 9.55)	2.13 (0.91 to 4.98)	0.09
Dominant inheritance model					
II	06	10	Ref	Ref	
DD + DI	14	87	3.72 (1.17 to 11.88)	2.70 (1.21 to 6.0)	0.026
Recessive inheritance model					
II + DI	10	50	Ref	Ref	
DD	10	47	0.94 (0.35 to 2.46)	0.95 (0.42 to 2.11)	0.89
Over dominant inheritance model					
II + DD	16	57	Ref	Ref	
DI	04	40	2.80 (0.87 to 9.025)	2.41 (0.86 to 6.75)	0.08

**Table 7:** Statistical comparisons (*p*-values) of ACE2-rs4646994 I/D genotypes with mortality of COVID-19 patients.

	N = 43	N = 74	Odd Ratio (95% CI)	Risk Ratio (95% CI)	*p*-Value
Codominant inheritance model					
II	11	05	Ref	Ref	
DI	20	24	2.64 (0.7854 to 8.8737)	1.51 (0.9524 to 2.4019)	0.11
DD	12	45	8.25 (2.40 to 28.34)	3.26 (1.7894 to 5.9596)	0.008
Dominant inheritance model					
II	11	05	Ref	Ref	
DD + DI	32	69	4.74 (1.5214 to 14.7915)	2.16 (1.4014 to 3.3599)	0.007
Recessive inheritance model					
II + DI	31	29	Ref	Ref	
DD	12	45	4.0 (1.7769 to 9.0432)	2.45 (1.4031 to 4.2927)	0.0008
Overdominant inheritance model					
II + DD	23	50	Ref	Ref	
DI	20	24	0.55 (0.2551 to 1.1946)	0.69 (0.4340 to 1.1070)	0.13

**Table 8:** ACE2 rs4240157 T > C gene polymorphism in COVID-19 patients and controls.

Subjects	N	TT%	CT%	CC%	fD	χ^2^	T	C	*p*-Value
Cases	117	41 (35)	47 (40.17)	29 (24.78)	2	14.34	0.55	0.45	0.0007
Controls	100	59 (59)	31 (31)	10 (10)			0.74	0.26	

**Table 9:** Multivariate analysis of ACE2 rs4240157 T > C gene polymorphism with COVID-19 severity.

Genotypes	Healthy Controls	COVID-19 Cases	OR (95% CI)	Risk Ratio (RR)	*p*-Value
	(N = 100)	(N = 117)			
Codominant					
ACE2-TT	59	41	1 (ref.)	1 (ref.)	
ACE2-CT	31	47	2.18 (1.92–3.99)	1.48(1.07–2.04)	0.01
ACE2-CC	10	29	2.66 (1.53–4.62)	1.68(1.25–2.26)	0.005
Dominant					
ACE2-TT	59	41	1 (ref.)	1 (ref.)	
ACE2-(CT + CC)	41	76	2.66 (1.53–4.62)	1.68(1.25–2.26)	0.005
Recessive					
ACE2-(TT + CT)	90	88	1 (ref.)	1 (ref.)	
ACE2-CC	10	29	2.96(1.36–6.44)	1.97 (1.13–3.43)	0.006
Allele					
ACE2-T	100	121	1 (ref.)	1 (ref.)	
ACE2-C	100	105	0.86 (0.59–1.27)	0.92 (0.75–1.13)	0.46

**Table 10:** Statistical comparisons (*p*-values) of ACE2 rs4240157 T > C genotypes with comorbid conditions and some clinical parameters with COVID-19 severity. T2D—type2 diabetes; CKD—chronic kidney disease; CAD—coronary artery disease; SpO_2_—partial pressure of oxygen; ALT—alanine transaminase; CRP—C reactive protein; AST—aspartate transaminase.

Clinical Feature	Variable	N	TT	CT	CC	fD	χ^2^	*p*
Age	<45	20	07	07	06	2	0.43	0.80
	>45	97	34	40	23			
Gender	Males	85	26	33	26	2	6.12	0.046
	Females	32	15	14	03			
T2D	Yes	47	11	27	09	2	9.88	0.007
	No	70	30	20	20			
CKD	Yes	11	04	04	03	2	0.08	0.960
	No	106	37	43	26			
Hypertension	Yes	37	14	16	07	2	1	0.006
	No	80	27	31	22			
CAD	Yes	17	05	11	01	2	6.03	0.049
	No	100	36	36	28			
Oxygen saturation	<60 mm Hg	47	14	13	20	2	13.34	0.001
	>80 mm Hg	70	33	28	09			
Duration in hospital	>30 days	57	17	26	14	2	1.69	0.420
	<30 days	60	24	21	15			
ALT	>36 units	45	19	21	05	2	7.36	0.025
	<36 units	72	22	26	24			
CRP	>0.8 mg	104	38	43	23	2	3.61	0.16
	<0.8 mg	13	03	04	06			
AST	>40	48	20	19	09	2	2.22	0.32
	<40	69	21	28	20			
Antiviral therapy	Yes	79	27	33	19	2	0.26	0.87
	No	38	14	14	10			
Steroids therapy	Yes	77	31	23	23	2	10.23	0.006
	No	40	10	24	06			
Survival	Death occurred	43	14	10	19	2	15.29	0.0005
	Survived	74	27	37	10			

**Table 11:** Statistical comparisons (*p*-values) of ACE2 rs4240157 T > C genotypes with mortality of COVID-19 patients.

Codominant Model	N = 74	N = 43	Odd Ratio (95% CI)	Risk Ratio (95% CI)	*p*-Value
TT	27	14	Ref	Ref	
CT	37	10	0.52 (0.20 to 1.34)	0.83 (0.64 to 1.09)	0.17
CC	10	19	3.66 (1.34 to 9.97)	1.90 (1.10–3.30)	0.011
Dominant model					
TT	27	14	Ref	Ref	
CC + CT	47	29	1.19 (0.5378 to 2.6332)	1.06 (0.8028 to 1.4124)	0.66
Recessive model					
TT + CT	64	24	Ref	Ref	
CC	10	19	5.06 (2.0640–12.437)	2.10 (1.2567 to 3.53)	0.004
Allele					
T	91	38	Ref	Ref	
C	57	48	2.01 (1.1761–3.45)	1.29 (1.05 to 1.59)	0.010

**Table 12:** Statistical comparisons (*p*-values) of ACE2 rs4240157 T > C genotypes in COVID-19 patients with various comorbidities.

Type 2 Diabetes					
Codominant Model	N = 70	N = 47	Odd Ratio (95% CI)	Risk Ratio (95% CI)	*p*-Value
TT	30	11	Ref	Ref	
CT	20	27	3.68 (1.495 to 9.064)	1.71 (1.175 to 2.515)	0.004
CC	20	09	1.22 (0.430 to 3.496)	1.06 (0.780 to 1.441)	0.070
Dominant model					
TT	30	11	Ref	Ref	
CC + CT	40	36	2.45 (1.076 to 5.599)	1.39 (1.048 to 1.844)	0.032
Allele					
T	70	47	Ref	Ref	
C	60	45	1.11 (0.654 to 1.906)	1.04 (0.838 to 1.307)	0.068
Hypertension					
Codominant model	N = 80	N = 37	Odd Ratio (95% CI)	Risk Ratio (95% CI)	*p*-value
TT	27	14	Ref	Ref	
CT	31	16	0.99 (0.4114 to 2.4084)	0.99 (0.7387 to 1.3495)	0.99
CC	22	07	0.61 (0.2110 to 1.7850)	0.86 (0.642 to 1.173)	0.370
Dominant model					
TT	27	14	Ref	Ref	
CC + CT	53	23	0.83 (0.3723 to 1.8814)	0.94 (0.7241 to 1.2315)	0.67
Coronary artery disease					
Codominant model	N = 100	N = 17	Odd Ratio (95% CI)	Risk Ratio (95% CI)	*p*-value
TT	36	05	Ref	Ref	
CT	36	11	2.20 (0.694 to 6.973)	1.14 (0.943 to 1.393)	0.180
CC	28	1			
Dominant model					
TT	36	05	Ref	Ref	
CC + CT	64	12	0.25 (0.028 to 2.328)	0.90 (0.796 to 1.039)	0.22
Age					
Codominant model	<45 N = 20	>45 N = 97	Odd Ratio (95% CI)	Risk Ratio (95% CI)	*p*-value
TT	20	34	Ref	Ref	
CT	07	40	3.36 (1.268 to 8.908)	2.48 (1.155 to 5.353)	0.014

**Table 13:** Distribution of ACE2- II, DI and DD genotypes in the SARS-CoV-2 patients in the world populations.

Country	Sample Size (N)	DD Genotype	% DD	ID Genotype	%ID	II Genotype	%II
USA	4320	1331	30.81	2145	49.65	844	19.54
UK	10566	2896	27.41	5241	49.60	2429	22.99
Canada	299	85	28.43	149	49.83	65	21.74
Turkey	672	238	35.42	303	45.09	131	19.49
Australia	444	134	30.18	216	48.65	94	21.17
Austria	2251	619	27.50	1108	49.22	524	23.28
Belgium	3980	1074	26.98	2030	51.01	876	22.01
India	660	130	19.70	316	47.88	214	32.42
China	3764	1154	30.66	1228	32.62	1382	36.72
Czech	443	116	26.19	225	50.79	102	23.02
Germany	5047	1345	26.65	2581	51.14	1121	22.21
Japan	379	68	17.94	185	48.81	126	33.25
Netherlands	6110	1705	27.91	3056	50.02	1349	22.08
South-Africa	300	46	15.33	158	52.67	96	32.00
Israel	87	9	10.34	50	57.47	28	32.18
Korea	785	256	32.61	294	37.45	235	29.94
Denmark	126	41	32.54	43	34.13	42	33.33
Italy	3151	1185	37.61	1358	43.10	608	19.30
Russia	320	126	39.38	125	39.06	69	21.56
Spain	1665	630	37.84	767	46.07	268	16.10
Iran	200	75	37.50	96	48.00	29	14.50
Turkey	90	31	34.44	14	15.55	45	50
Finland	115	49	42.61	52	45.22	14	12.17
Brazil	104	51	49.04	33	31.73	20	19.23
Saudi Arabia [current study]	117	57	48.71	44	37.60	16	13.67
